# Effects of Single Vs. Multiple Sets Water-Based Resistance Training on Maximal Dynamic Strength in Young Men

**DOI:** 10.1515/hukin-2015-0072

**Published:** 2015-10-14

**Authors:** Adriana Cristine Koch Buttelli, Stephanie Santana Pinto, Maira Cristina Wolf Schoenell, Bruna Pereira Almada, Liliana Kologeski Camargo, Matheus de Oliveira Conceição, Luiz Fernando Martins Kruel

**Affiliations:** 1 Physical Education School, Federal University of Rio Grande do Sul (UFRGS), Porto Alegre, Brazil.; 2 Physical Education School, Federal University of Pelotas (UFPel), Pelotas, Brazil.

**Keywords:** aquatic exercises, volume, maximal dynamic strength

## Abstract

The aim of this study was to compare the effects of single vs. multiple sets water-based resistance training on maximal dynamic strength in young men. Twenty-one physically active young men were randomly allocated into 2 groups: a single set group (SS, n=10) and a multiple sets group (MS, n=11). The single set program consisted of only 1 set of 30 s, whereas the multiple sets comprised 3 sets of 30 s (rest interval between sets equaled 1 min 30 s). All the water-based resistance exercises were performed at maximal effort and both groups trained twice a week for 10 weeks. Upper (bilateral elbow flexors and bilateral elbow extensors, peck deck and inverse peck deck) as well as lower-body (bilateral knee flexors and unilateral knee extensors) one-repetition maximal tests (1RM) were used to assess changes in muscle strength. The training-related effects were assessed using repeated measures two-way ANOVA (α=5%). Both SS and MS groups increased the upper and lower-body 1RM, with no differences between groups. Therefore, these data show that the maximal dynamic strength significantly increases in young men after 10 weeks of training in an aquatic environment, although the improvement in the strength levels is independent of the number of sets performed.

## Introduction

Water-based exercises comprise a series of specific movements that use water resistance to generate an overload. These exercises have been the subject of several studies in recent years and the results indicate countless benefits in both young adults (Colado et al., 2009a; Pinto et al., 2014) and elderly people (Colado et al., 2009b, 2012; Graef et al., 2010; Sanders et al., 2013). Water-based exercises promote improvements in the cardiovascular condition (Pinto et al., 2014; Takeshima et al., 2002; Taunton et al., 1996), body composition (Colado et al., 2012; Gappmaier et al., 2006; Takeshima et al., 2002), flexibility (Takeshima et al., 2002), balance (Elbar et al., 2012; Sanders et al., 2013) and muscle strength (Ambrosini et al., 2010; Colado et al., 2009a; Graef et al., 2010; Petrick et al., 2001; Pinto et al., 2014; Pöyhönen et al., 2002; Souza et al., 2010; Takeshima et al., 2002; Tsourlou et al., 2006).

Regarding the effects of resistance training in the aquatic environment, Souza et al. (2010) showed that 11 weeks of water-based circuit resistance training improved the upper and lower limbs maximal dynamic strength (i.e., one-repetition maximal test) in untrained young women. Similarly, Colado et al. (2009a) reported a significant increase in upper limb maximal dynamic strength (i.e., submaximal test to estimate the one-repetition maximal value) after short-term (i.e., 8 weeks) water-based resistance training in fit young men. Pöyhönen et al. (2002) showed an increase in the isometric and isokinetic extension and flexion torques, respectively, after 10 weeks of water-based resistance training in physically active women. Moreover, Pinto et al. (2014) also demonstrated that water-based concurrent training during 12 weeks improved both maximal dynamic and isometric strength of upper and lower limbs in untrained young women.

Regarding the volume of training, the goal of studies that compared a single set with multiple sets is to evaluate whether different amounts of training produce similar increases in muscle strength. In land-based resistance training, some studies (Bottaro et al., 2009; Hass et al., 2000; Kelly et al., 2007; Kraemer et al., 2000; Marx et al., 2001; McBride et al., 2003; Ronnestad et al., 2007; Schlumberger et al., 2001) have compared programs consisting of different training volumes in young subjects. With the objective of ascertaining whether increasing the training volume (from 1 to 3 sets) would result in muscle strength improvement, Hass et al. (2000) analyzed the effects of 13-weeks of the resistance training circuit and showed that muscle strength increased in a similar fashion (8 to 14%) in adults (recreational weightlifters) who performed 1 or 3 sets. In contrast, while analyzing a longer period of training in collegiate female tennis players, Kraemer et al. (2000) observed that multiple sets provided continuity to muscle strength gains throughout the training period (9 months), while a single set increased strength levels only during the first few weeks. However, to the authors’ knowledge, no studies have compared the effects of different training volumes in the aquatic environment.

The present study was designed in order to improve the knowledge regarding the effects of different training volumes on muscle strength increases during resistance training performed in the aquatic environment. Thus, the purpose of this study was to compare the effects of single and multiple sets water-based resistance training on maximal dynamic strength in active young men. Our hypothesis was that different resistance training volumes would exhibit similar strength gains in young men.

## Material and Methods

### Experimental design

To investigate the hypothesis of this study, we compared the effects of water-based resistance training at different volumes (i.e., single vs. multiple sets) on maximal dynamic strength in active young men. Both single and multiple sets groups trained for 10 weeks, and each subject was evaluated before and after (weeks 0 and 11) water-based resistance training. The post-measurements started 72 h after the last training session, and the subjects completed all the evaluations within a week with an interval of 48 h between the tests. Different tests were conducted on different days to prevent fatigue. To analyze the stability and reliability of the variables, eight subjects (21 ± 2.27 years) were evaluated twice before the start of training (weeks −4 and 0), which served as a control period for maximal dynamic strength. Among the eight subjects who participated in the control period, three were allocated to a single set and five to a multiple sets group.

### Participants

Twenty-one physically active and healthy young men, who were not engaged in any regular and systematic resistance training program in the previous 6 months, volunteered for the study after providing an informed consent form. The subjects volunteered for the present investigation following announcements in a widely read local newspaper. Exclusion criteria included any history of neuromuscular, metabolic, hormonal and cardiovascular diseases. Subjects were not taking any medication that could influence hormonal and neuromuscular metabolism and were advised not to change their nutrition practices throughout the study. Ten subjects were Physical Education students and they reported to perform different physical activities during the week. Subjects were carefully informed about the design of the study and the possible risks and discomforts related to the measurements. The research was approved by the Ethics Committee of the Federal University of Rio Grande do Sul, Brazil, and it was in accordance with the ethical standards laid down in the 1964 Declaration of Helsinki. The subjects were randomly assigned into two groups: single set (n=10) and multiple sets (n=11). Two participants abandoned the intervention due to personal reasons. Therefore, 19 volunteers completed the full study schedule.

### Body composition

Body mass and height were measured using an Asimed analog scale (with accuracy of 0.1 kg) and an Asimed stadiometer (with accuracy of 1 mm), respectively. Body composition was assessed using the skinfold technique. A 7-site skinfold equation was used to estimate body density (Jackson and Pollock, 1978), and body fat was subsequently calculated using the Siri equation (1993). In addition, the body mass index was calculated for each subject.

### Maximal strength

Maximal strength was assessed using the one-repetition maximal test (1RM) on the bilateral knee flexors and unilateral knee extensors (World-Esculptor, Porto Alegre, Brazil), bilateral elbow flexors (free-weight barbell) and bilateral elbow extensors (World-Esculptor, Porto Alegre, Brazil), peck deck and inverse peck deck (World-Esculptor, Porto Alegre, Brazil). The 1RM value was considered as the maximal load that could be exerted at the concentric phase for a given exercise. One week prior to the test day, subjects were familiarized with all procedures in 2 sessions. On the test day, the subjects warmed up for 5 min on a cycle ergometer, stretched all major muscle groups, and performed specific movements for the exercise test. Each subject’s maximal load was determined with no more than 5 attempts with a 2–3 min recovery between attempts. Performance time for each contraction (concentric and eccentric) was 2 s, controlled by an electronic metronome (MA-30, KORG, Tokyo, Japan). The 1RM was measured during two days (i.e., three exercises each day) with an interval of 48 h and all 1RM procedures were previously described by Cadore et al. (2010). The test–retest reliability coefficient (intraclass correlation coefficient, ICC) was 0.93 for the knee flexors 1RM and 0.69 for the knee extensors 1RM, 0.95 for the elbow flexors 1RM and 0.95 for the elbow extensors 1RM and 0.99 for the peck deck 1RM and 0.96 for the inverse peck deck 1RM. Each specific test at pre- and post-intervention was supervised by the same investigator, who was blinded to the training group of the subjects. The tests were conducted with the same equipment and identical subject/equipment positioning.

### Water-based resistance training

The sample followed a callisthenic (non-use of aquatic devices) water-based exercise program during 10 weeks. Subjects of the study trained 2 times a week, on nonconsecutive days. Training groups were differentiated by the exercise volume during the water-based resistance training. One group performed a single set (SS - 1 set of 30 s) and the other one performed multiple sets (MS - 3 sets of 30 s) of each resistance exercise. The total time per session spent by SS and MS was 25 and 50 min, respectively. Before the start of the water-based resistance training, subjects completed 2 familiarization sessions in the water environment to practice the exercises they would further perform during the training period as described by Pinto et al. (2014). Moreover, instructions about the rating of perceived exertion (RPE) scale were explained to the participants (Borg, 1990). The subjects performed the water-based resistance exercises progressively at all effort levels to familiarize themselves with the minimal effort and progression until they could reach maximal effort at their own pace.

The water-based resistance training was composed by upper and lower limbs exercises and by exercise for the abdominal musculature. The resistance exercises were divided into a circuit in both SS and MS groups. The pool was divided into four stations, and in each one the subjects performed 3 exercises, 12 exercises in total. After the performance of a set of each of the 3 exercises, an active rest interval of 1 min 30 s was taken, in which the subjects kept a stationary running exercise at an intensity equivalent to the perception 9 of the Borg scale (Borg, 1990). After the rest interval, the subjects of the SS group changed stations while the subjects of the MS group repeated the sequence of each station 3 times. The exercises used in the water-based resistance training are described in Table 1.

The exercises performed during the warm-up (i.e., 5 min) and the cool-down (i.e., 8 min) were standardized to avoid any possible interference with the aims of the study. Two experienced water-based instructors carefully supervised all training sessions. During the resistance exercises, the individuals were instructed to perform each repetition at maximal effort and amplitude in order to achieve the greatest velocity of motion as possible, and consequently, greater resistance. Verbal encouragement was provided during all resistance training sessions by the same instructor. Throughout the training period, the water temperature was maintained at 31.0 ± 0.1 °C. In addition, the water depth for each subject was fixed between the xiphoid process and shoulders.

### Statistical analysis

Results are reported as mean ± SD. Normal distribution and homogeneity parameters were checked with Shapiro-Wilk and Levene tests, respectively. Statistical comparisons in the control period (from weeks −4 to 0) were performed by using Student paired t tests. In addition the test-retest reliability for each 1RM test between the weeks −4 to 0 was determined using the intraclass correlation coefficient (ICC). The training-related effects were assessed using repeated measures 2-way ANOVA (factors: group and time). Significance was accepted when α=5%, and the SPSS statistical software package (version 17.0) was used to analyze all data.

## Results

The demographic characteristics of the remaining subjects (SS n=10; MS n=9) included in the data analysis were as follows (mean ± SD): SS age 22 ± 3.8 years, body mass 76.5 ± 13.4 kg, body height 1.8 ± 0.1 m, percent fat 24.7 ± 16.4%, body mass index 24.7 ± 4 kg/m^2^; MS age 21.4 ± 3 years, body mass 78.4 ± 11.2 kg, body height 1.8 ± 0.1 m, percent fat 18.9 ± 11.3%, body mass index 24.3 ± 3.8 kg/m^2^.

During the control period (weeks −4 and 0), no changes were observed in most variables analyzed (Table 2). However, slight changes were found for the elbow extensors, knee flexors and inverse peck deck 1RM between weeks −4 and 0. No significant differences were observed in training compliance between SS and MS (91.36 ± 8.69 vs. 90.40 ± 8.33%, respectively). In addition, there were no differences between groups before training in age (p=0.729), body mass (p=0.747), body height (p=0.203), percent fat (p=0.386) and body mass index (p=0.790).

At baseline, there were no differences between groups in bilateral knee flexors and unilateral knee extensors 1RM, bilateral elbow flexors and bilateral elbow extensors 1RM, as well as peck deck and inverse peck deck 1RM. After training, there were increases in bilateral knee flexors (SS: 12.30 ± 7.82 vs. MS: 11.00 ± 6.58%) and unilateral knee extensors 1RM (SS: 9.60 ± 10.86 vs. MS: 9.51 ± 6.57%), bilateral elbow flexors (SS: 5.10 ± 5.51 vs. MS: 4.79 ± 5.42%), bilateral elbow extensors 1RM (SS: 4.79 ± 9.99 vs. MS: 7.98 ± 4.23%), peck deck (SS: 3.41 ± 5.50 vs. MS: 5.58 ± 4.01%) and inverse peck deck 1RM (SS: 7.73 ± 9.75 vs. MS: 6.50 ± 8.06%) in both SS and MS, with no statistically significant difference between groups. These results are described in Table 3.

## Discussion

The primary finding of this study was that the training volume was not a determining factor for greater muscle strength increments, since the SS group exhibited similar strength gains compared to the MS group.

The results of this study demonstrated that young men after 10 weeks of training in an aquatic environment increased maximal dynamic strength of upper and lower limbs. These data are in agreement with some previous studies on resistance training in the aquatic environment (Colado et al., 2009a; Pinto et al., 2014). In the only previous study (Colado et al., 2009a) that assessed 8 weeks of water-based resistance training in young men a 5% increase in muscle strength on the flat bench press was found. The results of the present study are in agreement with the study of Colado et al. (2009a), since we found 3 and 5% increases in 1RM of peck deck in the SS and MS group, respectively. Similarly, Souza et al. (2010) analyzed the effects of 11 weeks of water-based resistance training on maximal dynamic strength of several muscle groups in untrained young women. In support of the results of the present study, these authors observed significant increases in muscle strength for all exercises assessed (i.e., bench press: 25%, knee extension: 20%; knee flexion: 17% and seated row: 12%). However, the goal of the previous studies was not to compare the volume of training on muscle strength, as the period of training was divided into different mesocycles. Thus, the differences observed between these strength gains percentages may be explained by the periodization model adopted in the present study, where the volume was kept constant, as our goal was to assess the different effects of training using 1 or 3 sets. Moreover, the effects of resistance training depend on subjects’ training backgrounds (i.e., untrained young women vs. physically active young men).

In the literature reviewed, no studies comparing different training volumes in the aquatic environment were found; only land-based studies have been performed to address this subject. Thus, a comparison with land-based studies was deemed necessary. Previous studies have shown (Cannon and Marino, 2010; Hass et al., 2000; Kraemer et al., 2000; Radaelli et al., 2013; Starkey et al., 1996) that SS can be considered an alternative to MS training, as this approach saves time and generates similar strength increments. Some of these studies were included in the meta-analysis performed by Fröhlich and Emrich (2010), which identified 72 studies published between 1985 and 2008. The results of this meta-analysis support those of the present research, as the authors stated that for short-term interventions, such as the 10-week duration of this study, the effects of training were identical for both groups. However, for long-term observations, MS training may be suggested for trained individuals who seek additional muscle strength gains. Nevertheless, the study developed by Schlumberger et al. (2001) showed that short-term intervention (i.e., 6-week) presented greater percentage strength gains for the bilateral leg extension 1RM in women with basic experience in resistance training (3–6 months) in the multiple sets group compared to the single set resistance training group (15% vs. 6%).

According to Hass et al. (2001), additional sets do not produce significant improvements in muscle strength after 13 weeks of training using land-based circuits in adult recreational weight lifters. The aim of this previous study was to confirm whether increasing the training volume (from 1 to 3 sets) would result in increases in muscle strength, and the results showed a similar increase in muscle strength for leg extension (14% vs. 13%), leg curl (9% vs. 12%) and chest press (12% vs. 13%) among subjects whose training included 1 or 3 sets. Also similar to this previously cited study, McBride et al. (2003) concluded that untrained individuals (men and women) who trained for 1 set and those who trained for 6 sets presented similar percentage gains in muscle strength on leg press exercise after 12 weeks of training (41% vs. 53%). The results of the present study are in line with the aforementioned studies, since similar percentage gains in muscle strength were found in fit young men that trained 1 or 3 sets in water-based resistance training (i.e., bilateral knee flexors 1RM SS: 12% vs. MS: 11%; unilateral knee extensors 1RM SS: 10% vs. MS: 9%; peck deck 1RM SS: 3% vs. MS: 5%). However, Marx et al. (2001) found that the high-volume training (MS) was superior to the low-volume training (SS) for muscle performance increases in non-trained women. As the training duration is increased beyond 10 weeks, the dynamics of neural and hypertrophic relationships are inverted, i.e., muscle hypertrophy is predominant, and its contribution to increases in strength and power becomes greater than that of neural factors. Therefore, the difference in these results may have been due to the longer period of training, i.e., 24 weeks, as compared to 10 weeks in the present study.

A possible limitation of the present study was that the participants performed a water-based resistance training composed of sets lasting 30 s, which could induce greatest changes in muscle endurance, and only maximal strength was assessed in this study. Thus, it is possible that differences between single vs. multiple sets water-based resistance training could occur if a specific test for muscle endurance would be performed, which remains speculative. In addition, the sample size could be a limitation, since only 10 young men composed the single set group and 9 the multiple sets group.

In summary, the results of the present study showed that the upper and lower limbs maximal dynamic strength significantly increased in young men after 10 weeks of water-based resistance training. In addition, the improvement in the strength levels was independent of the number of sets performed (i.e., single vs. multiple sets). Regarding the practical importance of our findings, we found that single or multiple sets water-based resistance training in young active men exhibited similar maximal dynamic strength increases in upper and lower limbs. If a subject focuses on aerobic exercises (i.e., weight reduction), single set water-based resistance training may be a time-efficient supplement for increasing strength. Moreover, a short training session composed by only 1 set per exercise may help to avoid a high dropout rate in water-based resistance training when subjects are unwilling to perform longer resistance training sessions.

## Figures and Tables

**Table 1:** Single (SS) and multiple sets (MS) water-based resistance training

Sets/Duration	Exercise name	Description of the joint movements
Station 1 SS 1 set 30 s MS 3 sets 30 s	Hip adduction/abduction	Hip adduction and abduction (simultaneously).
	Horizontal shoulder flexion/extension	Horizontal shoulder flexion and extension (simultaneously).
	Lateral crunch	Trunk lateral flexion and extension in horizontal position, with a noodle supporting the back (alternately left and right side).
Station 2 SS 1 set 30 s MS 3 sets 30 s	Frontal kick and slide back with the right leg	The first phase of the movement consisted of a right hip flexion at 90°, with right knee extension; the second phase consisted of a right hip and knee extension.
	Frontal kick and slide back with the left leg	The first phase of the movement consisted of a left hip flexion at 90°, with left knee extension; the second phase consisted of a left hip and knee extension.
	Frontal crunch 2-times	Trunk flexion and extension in horizontal position performed in 2-times, with a noodle supporting the back.
Station 3 SS 1 set 30 s MS 3 sets 30 s	Knee flexion/extension with the right leg	Right knee flexion and extension (starting from hip flexion at 90°).
	Knee flexion/extension with the left leg	Left knee flexion and extension (starting from hip flexion at 90°).
	Shoulder adduction/abduction	Shoulder adduction and abduction (simultaneously).
Station 4 SS 1 set 30 s MS 3 sets 30 s	Elbow flexion/extension with the right arm	Right elbow flexion and extension (starting from the anatomical position).
	Elbow flexion/extension with the left arm	Left elbow flexion and extension (starting from the anatomical position).
	Frontal crunch	Trunk flexion and extension performed in the horizontal position, with a noodle supporting the back.

Interval among stations and/or between sets was 1 min 30 s.

**Table 2:** Pre and post values during the control period (−4 and 0 weeks)

	Subjects n=8				

	Week −4		Week 0		

	Mean	SD	Mean	SD	p
EFLE 1RM (kg)	32.37	±4.50	34.00	±5.34	0.075
EEX 1RM (kg)	55.50	±7.78	59.87	±8.44	<0.001*
KFLE 1RM (kg)	65.12	±10.64	67.12	±11.85	0.025*
KEX 1RM (kg)	68.37	±7.42	66.62	±9.16	0.578
PD 1RM (kg)	54.00	±11.98	52.87	±10.89	0.450
IPD 1RM (kg)	63.75	±9.47	67.12	±11.93	0.038*

EFLE and EEX 1RM, elbow flexors and elbow extensors one maximal repetition; KFLE 1RM and KEX 1RM, knee flexors and knee extensors one maximal repetition; PD 1RM and IPD 1RM, peck deck and inverse peck deck one maximal repetition.

* indicate significant difference between week −4 and 0.

**Table 3:** Strength performance before and after training: water-based single set and water-based multiple sets; mean ±SD

	Single Set, n=10				Multiple Sets, n=9						

	Pre		Post		Pre		Post		Group	Time	Group*Time

	Mean	SD	Mean	SD	Mean	SD	Mean	SD	p	p	p
EFLE 1RM (kg)	31.40	±5.08	32.80	±4.39	35.67	±5.17	37.22	±4.66	0.064	<0.001*	0.820
EEX 1RM (kg)	58.40	±11.13	60.50	±9.28	64.33	±8.99	69.33	±8.87	0.108	0.001*	0.104
KFLE 1RM (kg)	62.60	±12.84	70.10	±13.57	67.11	±8.78	74.44	±10.14	0.134	0.001*	0.143
KEX 1RM (kg)	67.90	±9.48	74.40	±12.70	67.89	±9.31	74.37	±11.59	0.133	0.001*	0.755
PD 1RM (kg)	50.40	±13.32	51.90	±13.08	58.33	±10.58	61.67	±11.87	0.994	<0.001*	0.985
IPD 1RM (kg)	63.80	±14.24	68.60	±13.13	73.22	±12.72	77.33	±10.32	0.410	<0.001*	0.932

EFLE and EEX 1RM, elbow flexors and elbow extensors one maximal repetition; KFLE 1RM and KEX 1RM, knee flexors and knee extensors one maximal repetition; PD 1RM and IPD 1RM, peck deck and inverse peck deck one maximal repetition.

* indicate significant difference between pre and post training in both groups (single and multiple sets).
